# Profil épidemio-clinique et radiologique des atteintes ostéo-articulaires des hémophiles à Madagascar

**DOI:** 10.11604/pamj.2014.19.287.5237

**Published:** 2014-11-15

**Authors:** Lova Hasina Rajaonarison Ny Ony Narindra, Feno Hasina Rabemanorintsoa, Faralahy Ravelonarivo Randrianantenaina, Olivat Alson Aimée Rakoto, Ahmad Ahmad

**Affiliations:** 1 Service Imagerie Médicale, Hôpital Universitaire Joseph Ravoahangy Andrianavalona, Ampefiloha, Antananarivo, Madagascar; 2 Service Biologie-Hématologie, Hôpital Universitaire Joseph Ravoahangy Andrianavalona, Antananarivo, Madagascar

**Keywords:** Hémophile, Madagascar, radiologie, échographie, Hemophiliac, Madagascar, radiology, ultrasound

## Abstract

**Introduction:**

Déterminer le profil épidémio-clinique et radiologique des atteintes ostéo-articulaires des hémophiles malagasy.

**Méthodes:**

Une étude prospective, descriptive portant sur 25 patients hémophiles venant de tout Madagascar a été réalisée. Des radiographies numérisées des genoux, des chevilles et des coudes en incidence de face et de profil ainsi qu'une échographie des hanches, des genoux, des chevilles et des coudes ont été réalisées chez ces patients. Le type et la sévérité de la maladie ainsi que l'aspect de la cavité articulaire, la synoviale, les noyaux épiphysaires et les surfaces articulaires ont été analysés.

**Résultats:**

Soixante-huit pourcent des patients étaient hémophiles de type A et 32 % de type B. Quarante pourcent étaient classés sévères, 28 % modérés et 32 % mineurs. Les atteintes ostéo-articulaires ont été retrouvées chez 56 % des patients. Il n'existait pas de prédominance d'atteinte selon le type ni la sévérité de la maladie. Les plus jeunes étaient les plus atteints et l'articulation du genou et de la cheville étaient les plus touchées.

**Conclusion:**

Les complications ostéo-articulaire de l'hémophilie sont graves et ne dépendent pas du type ni de la sévérité de l'affection. Elles touchent surtout les enfants d'âge scolaire. Le couple radiographie-échographie permet de diagnostiquer et de surveiller ces lésions.

## Introduction

L´hémophilie est une maladie héréditaire à transmission récessive liée au chromosome X. C´est une pathologie rare mais grave caractérisée par un déficit en facteur de coagulation. Il existe deux formes: l´hémophilie ‹‹A›› caractérisée par un déficit en facteur VIII avec une fréquence de 1 sur 10 000, et l´hémophilie ‹‹B›› par un déficit en facteur IX qui est cinq fois moins fréquente [1]. Le diagnostic est biologique. L´imagerie est utile pour la détection et la surveillance des complications notamment ostéo-articulaires. Les objectifs de cette étude sont de déterminer le profil épidémiologique et d´évaluer les aspectsradiologiques des atteintes ostéo-articulaires des hémophiles à Madagascar.

## Méthodes

Il s'agit d´une étude prospective de trois mois s´étalant du 30 janvier au 30 Mars 2012, réalisée dans le service d'Imagerie Médicale et de Biologie-Hématologie de l'Hôpital Universitaire Joseph Ravoahangy Andrianavalona portant sur 25 patients hémophiles confirmés et recensés sur Madagascar par le service d'Hématologie. Nous avons réalisé une radiographie conventionnelle numérisée en incidence de face et de profil des grosses articulations intéressant les coudes, les genoux et les chevilles ainsi qu´une échographie des coudes, des hanches, des genoux et des chevilles de ces patients. L'analyse est portée sur la présence ou non d´épanchement dans les cavités articulaires, l'état de la synoviale, l´aspect de la corticale osseuse, du noyau épiphysaire et des surfaces articulaires. Nous avons recueillis les dossiers biologiques des patients afin de connaitre le type de l'affection et la sévérité de l'atteinte par le taux de facteur de coagulation manquant.

## Résultats

### Type d'hémophilie

Nous avons colligé 17 cas soit 68 % d'hémophile de type A contre 08 cas soit 32 % de type B. Dix (10) soit 40 % des patients sont hémophiles sévères, 07 soit 28 % sont hémophiles modérés et 08 soit 32 % sont hémophiles mineurs.

### Répartition des atteintes ostéo-articulaires en fonction de l'âge

L'âge moyen des patients était de 12 ans avec des extrêmes de 3 ans et 36 ans. Le tranches 0 à 10 ans avaient des complications ostéo-articulaires dans 24%, les 11 à 20 ans dans 20%, le 21 à 30 ans dan 8% et les 31 à 40 dans 4%.

### Atteintes ostéo-articulaires selon le type et la sévérité de l'hémophilie (Tableau 1 et Tableau 2)

**Tableau 1:** Type de l'hémophilie et atteintes ostéo-articulaires

Type		Hémophile sévère	Hémophile modéré	Hémophile mineure	Lésions ostéo-articulaires
**A**		24 %	20 %	24 %	32 %
**B**		16 %	8 %	8 %	24 %
**Total**		40 %	28 %	32 %	56 %

Note : hémophile mineure : F < 1% ; Hémophile modéré : 1% < F<5% ; Hémophile mineure : 5 < F<30% ; F : Facteur de coagulation

**Tableau 2:** Sévérité de l'hémophilie et atteintes ostéo-articulaires

Type		Sévérité	Atteintes ostéo-articulaires
**A**		sévère	4 (16 %)
		modéré	2 (08 %)
		mineur	2 (08 %)
		sévère	2 (08 %)
**B**		modéré	3 (12 %)
		mineur	1 (04 %)
**Total**			14 (56 %)

### Localisation des lésions (Tableau 3)

**Tableau 3:** Localisation des lésions ostéo-articulaires

Siège des lésions	Effectif	Pourcentage
Coude	4	17%
Hanche	1	4,34 %
Genou	10	43,47 %
Cheville	06	26, 06 %
Os long	02	8,69 %

### Types des lésions

**Figure 1:**
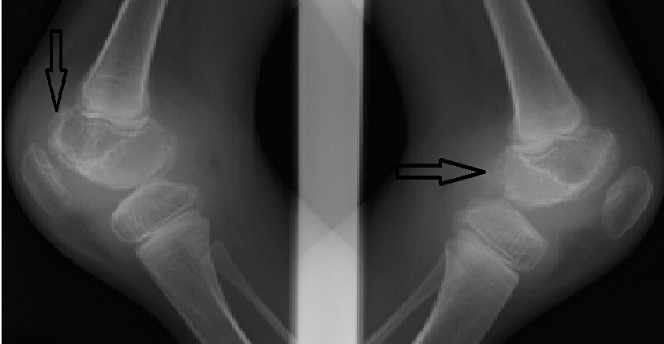
Enfant 15 ans, hémophile A sévère. Radiographie comparative en incidence de profil des genoux. Irrégularité des corticales osseuses condyliennes fémorales bilatérales (flèches). Noter l'hypertrophie épiphysaire et le gonflement des parties molles des deux genoux

**Figure 2:**
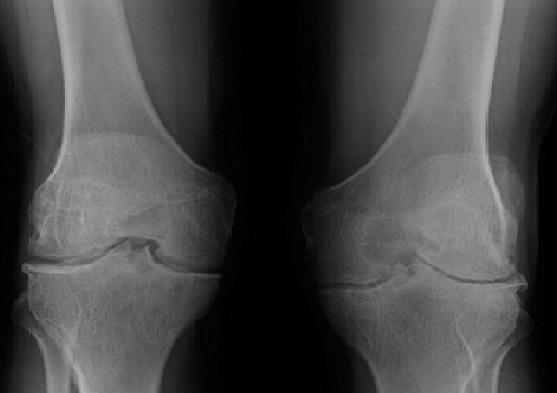
Homme 32 ans, hémophile B mineur. Radiographie comparative des genoux en incidence de face montrant des géodes des plateaux tibiaux et des condyles fémoraux associées à un épointement des épines tibiales et pincement de l'interligne fémoro-tibiale bilatérale: arthropathies évoluées

**Figure 3:**
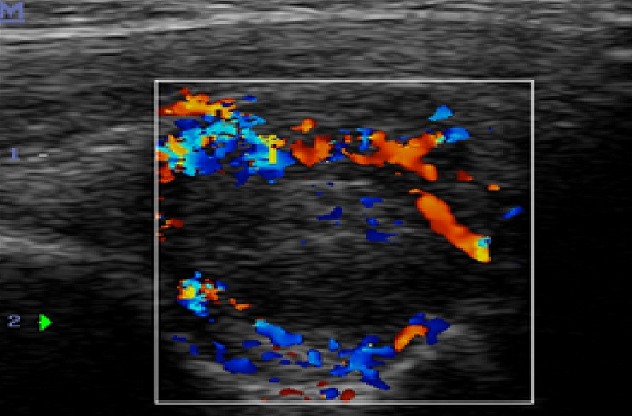
Enfant 08 ans, hémophile A modéré. Echographie conventionnelle en coupe sagittale de la face antérieure du coude gauche montrant un épaississement de la synoviale avec important flux vasculaire au codage Doppler et collection de liquide échogène intra-articulaire: hémarthrose avec synovite

**Figure 4:**
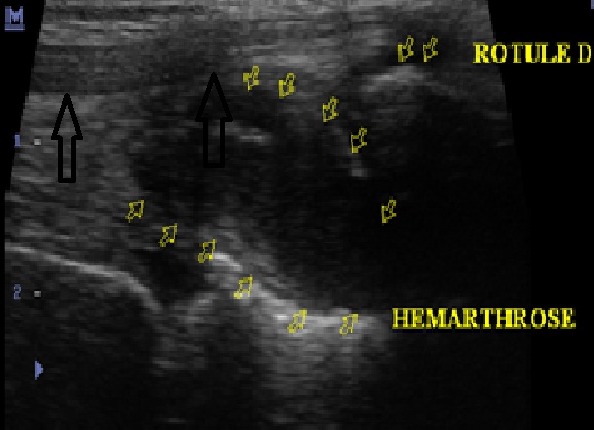
Enfant 08 ans, hémophile A modéré. Echographie de la face antérieure du genou droit. Structure liquidienne intra-articulaire (têtes de flèche) visible dans le récessus retro-quadricipital (flèches):hémarthrose

**Figure 5:**
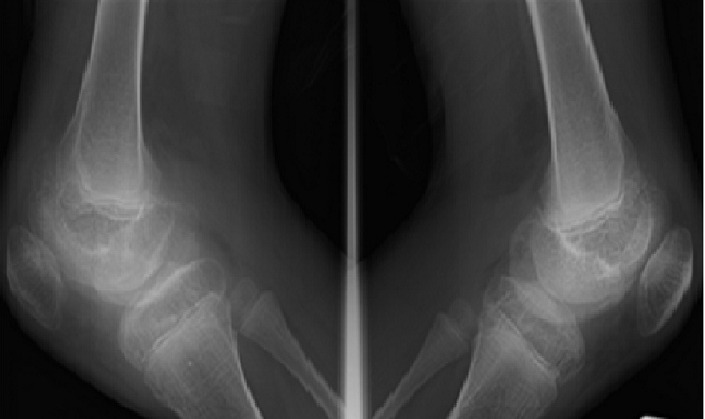
Enfant 08 ans, hémophile A sévère. Radiographie comparative des genoux en incidence de profil montrant une hypertrophie des noyaux épiphysaires condyliens fémoraux prédominant à droite

**Figure 6:**
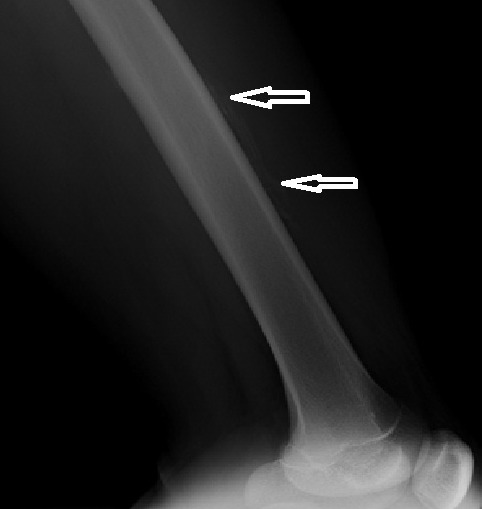
Garçon 16 ans, hémophile B sévère. Radiographie du fémur gauche en incidence de profil montrant des appositions périostées pluri-lamellaires (flèches) médio-diaphysaires

Une irrégularité de la corticale osseuse métaphyso-épiphysaire (Figure 1) a été observée dans 12 cas, suivi d'une atteinte des surfaces articulaires dans 10 cas (Figure 2). Les autres lésions étaient respectivement: l'épaississement synovial dans 6 cas (Figure 3), l'épanchement articulaire dans 5 cas (Figure 4) et une hypertrophie épiphysaire dans 4 cas (Figure 5). On notait également 03 cas d'atteinte diaphysaire des os longs à type d'ostéopénie, apposition périostée et des lignes d'arrêt de croissance (Figure 6).

## Discussion

Cette étude révèle la prédominance des hémophiles de type A (68 %) par rapport au type B (28 %) rapprochant les résultats de l'étude de Belhani avec une proportion de 85 % pour le type A sur 15 % pour le type B [2]. La maladie est dite sévère si le taux de facteur manquant est inférieur à 1% ; modérée entre 1% et 5 % et mineure entre 5 % et 30 % [1]. Quarantepourcent (40 %) de nos patients étaient de forme sévère, 28 % de forme modérée et 32 % de forme mineure. Ces résultats rejoignent ceux des études faites en Europe et aux Etats Unis [3, 4]. Ce qui n'est pas le cas pour Diop qui rapportait la prédominance des formes modérées suivies des formes majeures puis mineures qu'il estimait être liée à la forte mortalité des formes majeures et la difficulté diagnostique des formes mineures [5]. Il s'agit dans cette étude de population jeune avec un âge moyen de 12 ans et des âges extrêmes de 3 ans et 36 ans, ce qui pourrait être liée à l'espérance de vie des hémophiles à Madagascar où le diagnostic et la prise en charge restent encore peu accessibles.

En pays développé, l'espérance de vie des hémophiles atteint celle de la population normale, en l'absence de contamination par le virus du SIDA, grâce à l'évolution des thérapeutiques transfusionnelles [6]. Dans notre série, 56 % des patients,soit 32 % des types A et 24 % des types B, ont présentés des atteintes ostéo-articulaires. Pour Diop ces lésions sont présentes chez 53%des hémophiles [7]. Les plus jeunes ont été les plus touchés par ces atteintes ostéo-articulaires puisque, dans cette étude, 42,85 % des sujets atteints ont été âgés de 0 à 10 ans et 35,71% de 11 à 20 ans. Il s'agissait, pour ces jeunes, d'une irrégularité des surfaces articulaires et des épanchements intra-articulaires. Ceci s'expliquerait par le fait que la prévention des atteintes est encore assez difficile chez ces petits enfants qui comprendraient peu ou pas le mécanisme de cette maladie et notamment des atteintes ostéo-articulaires.

Nous avons remarqué, dans cette étude, qu'il n'y pas de proportionnalité entre la sévérité de l'hémophilie et l'atteinte ostéo-articulaire. Ainsi, 12 % d'hémophile B modéré ont présentés des atteintes ostéo-articulaires contre 8 % d'hémophile sévère du même type alors que 16 % d'hémophile sévère de type A ont eu ces lésions face à 8 % de forme modérée et 8 % de forme mineure de ce type A. Une étude menée à Dakar concluait la non influence de la sévérité de l´hémophilie sur la survenue des hématomes [7] alors que c'est l'hémarthrose répétée qui conduit aux lésions ostéo-articulaires chez l'hémophile [1]. Toutes les trois catégories de sévérité ont présenté les mêmes types de lésions donc l'hémophilie même mineurepeut être responsable d'atteinte ostéo-articulaire. Selon Klukowka [8] les aspects des atteintes ostéo-articulaires des hémophiles sont fortement lies aux nombres d'épisode d'hémarthrose. En effet la survenue d'une hémarthrose, facilement détectable en échographie, va entrainer des modifications synoviales, cartilagineuses et osseuses. L'atteinte au niveau du genou a prédominé dans notre série suivie des lésions de la cheville et du coude. D'après Kerr [9] les atteintes sont le plus souvent vues aux articulations les plus exposées aux traumatismes. Chez les hémophiles âgées plus de 30 ans, le genou et le coude sont les plus fréquemment lésés et chez les adolescents, c'est surtout la cheville. Selon la classification radiographique d'Arnold et Higartner, les lésions hémophiliques sont divisées en cinq stades [10] : Stade I: aucune anomalie radiologique; Stade II: arthropathie subaigüe caractérisée par une ostéopénie et un élargissement épiphysaire; Stade III: densification synoviale avec kystes sous chondraux articulaires et conservation de l'interligne articulaire; Stade IV: pincement de l'interligne articulaire avec majoration des lésions du stade III; Stade V: dislocation et désaxation articulaire.

Toutes ces lésions ont été retrouvées dans notre série de jeunes hémophiles sauf heureusement la dislocation et la désaxation articulaire. Par contre, les lignes d'arrêt de croissance visible surtout au niveau métaphyso-diaphysaire et la possibilité d'apposition périostée pluri-lamellaire ne figurant pas parmi cette classification restent à considérer. Le scanner ne présente pas d'intérêt particulier pour les arthropathies hémophiliques, mais l'imagerie par résonance magnétique (IRM) peut montrer précocement les altérations articulaires dès le premier épisode d'hémarthrose alors que la radiographie est encore normale [11]. Mais comme l'IRM est peu accessible ou difficilement réalisable surtout chez les petits enfants, la radiographie conventionnelle couplée à l'échographie reste une technique non négligeable dans le diagnostic et la surveillance des atteintes ostéo-articulaires chez les hémophiles.

## Conclusion

L'hémophilie est une pathologie rare. Elle peut donner des complications ostéo-articulaires graves secondaires à l'hémarthrose répétée liée au déficit en facteur de coagulation. Ces lésions se manifestent par des modifications synoviales, cartilagineuses et osseuses. Malgré l'évolution technologique de l'imagerie, le couple radiographie-échographie garde une place non négligeable dans le diagnostic et la surveillance de ces lésions hémophiliques.
